# A long non-coding RNA essential for early embryonic development improves somatic cell nuclear transfer somatic cell nuclear transfer efficiency in goats

**DOI:** 10.1530/REP-23-0053

**Published:** 2023-09-08

**Authors:** Miaomiao Jin, Lu Zhao, Hanwen Yang, Jianglin Zhao, Hongwei Ma, Yanzhi Chen, Jingcheng Zhang, Yan Luo, Yong Zhang, Jun Liu

**Affiliations:** 1 College of Veterinary Medicine, Northwest A&F University, Key Laboratory of Animal Biotechnology of the Ministry of Agriculture, Yangling, Shaanxi, China; 2 College of Animal Engineering, Yangling Vocational and Technical College, Yangling, Shaanxi, China

## Abstract

**In brief:**

Early embryonic development in goats is a complex and an important process. This study identified a novel long non-coding RNA (lncRNA), *lncRNA3720*, that appears to affect early embryonic development in goats through histone variants.

**Abstract:**

Although abundant lncRNAs have been found to be highly expressed in early embryos, the functions and mechanisms of most lncRNAs in regulating embryonic development remain unclear. This study was conducted to identify the key lncRNAs during embryonic genome activation (EGA) for promoting embryonic development after somatic cell nuclear transfer (SCNT) in goats. We screened and characterized lncRNAs from transcriptome data of *in vitro*-fertilized, two-cell (IVF-2c) and eight-cell embryos (IVF-8c) and eight-cell SCNT embryos (SCNT-8c). We obtained 12 differentially expressed lncRNAs that were highly expressed in IVF-8c embryos compared to IVF-2c and less expressed in SCNT-8c embryos. After target gene prediction, expression verification, and functional deletion experiments, we found that the expression level of *lncRNA3720* affected the early embryonic development in goats. We cloned full-length *lncRNA3720* and over-expressed it in goat fetal fibroblasts (GFFs). We identified histone variants by analyzing the transcriptome data from both GFFs and embryos. Gene annotation of the gene library and the literature search revealed that histone variants may have important roles in early embryo development, so we selected them as the potential target genes for *lncRNA3720*. Lastly, we compensated for the low expression of *lncRNA3720* in SCNT embryos by microinjection and showed that the development rate and quality of SCNT embryos were significantly improved. We speculate that *lncRNA3720* is a key promoter of embryonic development in goats by interacting with histone variants.

## Introduction

Terminally differentiated cells can be reprogramed into a totipotent state by somatic cell nuclear transfer (SCNT) which can be used to generate full-term mammals (Yang *et al.* 2007). Thus, SCNT has potential applications in agriculture, regenerative medicine and endangered species preservation (Liu *et al.* 2018). However, the extremely low cloning efficiency restricts the wider use of SCNT.

In recent years, many molecular mechanisms for abnormal development of cloned embryos have been discovered by using high-throughput sequencing. Numerous studies have reported that the factors affecting SCNT efficiency may include aberrant epigenetic modification in cloned embryos, such as DNA hypermethylation (Dean *et al.* 2001, Peat & Reik 2012), histone hypoacetylation (Iager *et al.* 2008) and abnormal histone methylation (Matoba *et al.* 2014, Liu *et al.* 2016*b*, Xie *et al.* 2016). Abnormal epigenetic modification results in abnormal gene expression and arrest of embryo development. It has been reported that the abnormal epigenetic modification was mainly concentrated in the non-coding sequences of the genome (Rinn *et al.* 2007, Matoba *et al.* 2014). Recently, emerging evidence has indicated that lncRNAs play an important role in early embryonic development by improving the reprograming efficiency of SCNT. Impeding *Xist* (X-inactive specific transcript) expression improved SCNT efficiency in mouse and pig (Matoba *et al.* 2014, Ruan *et al.* 2018). HPAT5, a primate-specific lincRNA, regulates pluripotency during human preimplantation development and nuclear reprograming (Durruthy-Durruthy *et al.* 2016). LincGET is essential for mouse embryonic development beyond the two-cell stage (Liu *et al.* 2016*a*). lncRNA 137 was also crucial for cleavage stage embryonic development in goat embryos (Han *et al.* 2018). Wu *et al.* revealed that lncRNAs potentially functioned synergistically in the cellular reprograming of SCNT embryos (Wu *et al.* 2018). In addition, the lncRNAs, TCONS_00166370 and TCONS_00020255, may play a vital role in porcine early embryogenesis (Li *et al.* 2016). Consequently, we speculated that lncRNAs may play a significant role in goat early embryos by facilitating reprograming in SCNT.

While EGA is critical for early embryonic development during the reprograming of SCNT, other genes involved include *MuERV-L*, *ZSCAN4*, *EIF1AX* and *Hsp07.1* (Shin *et al.* 2010, Liu *et al.* 2019*b*). Many lncRNAs were found to be closely associated with upregulation of their homologs in ZGA of mice (Hamazaki *et al.* 2015, Arlic *et al.* 2017). Histones are the primary proteins controlling gene expression; in addition to the canonical histones added during DNA replication, there are non-canonical histone variants. Histone variants can induce local epigenetic changes by modulating the structure and stability of nucleosomes or by recruiting variant-specific interacting proteins, thereby affecting chromatin accessibility (Buschbeck & Hake 2017). Among the histone H2A family, H2A.Z is involved in EGA (Ibarra-Morales *et al.* 2021) and gene regulation (Mylonas *et al.* 2021) in the nucleus and compensates for H3.3 during EGA to promote embryonic development (Shindo & Amodeo 2019).

In our previous study, we obtained lncRNA and mRNA expression profiles during EGA in goats by RNA sequencing and a set of lncRNAs highly expressed in eight-cell embryos (Deng *et al.* 2019). In this study, we analyzed the transcriptome of SCNT embryos and found abundant, highly expressed lncRNAs in IVF-8c embryos that were insufficiently activated in SCNT embryos. We identified a critical lncRNA (*lncRNA3720*) that promoted development of early embryos. Overexpression of *lncRNA3720* improved the developmental potential of goat SCNT embryos.

## Materials and methods

### Collection of oocytes and* in vitro* maturation

Goat ovaries were collected from a local abattoir and transported to our laboratory in sterile saline solution within 3-4 h at a controlled temperature of about 20°C. We opened the follicles (from 2 to 6 mm in diameter) with a sterile scalpel in phosphate-buffered saline (PBS) supplemented with 3% fetal bovine serum and collected the cumulus–oocyte complexes (COCs) of uniform size and with complete granulosa cells. The embryos were then washed three times with PBS and cultured in medium TCM-199 (Gibco, BRLGrand Island) supplemented with 10% FBS, 0.2 mmol/L sodium pyruvate, 0.075 IU/mL human menopausal gonadotropin, 1 μg/mL 17β-estradiol, 10 ng/mL epidermal growth factor, and 1% insulin–transferrin–selenium for 22–24 h under the conditions of 38.5°C in 5% CO_2_. After IVM, COCs were incubated in PBS with 0.1% bovine testicular hyaluronidase, and cumulus cells were gently removed by pipetting. Oocytes with evenly granulated ooplasms and first polar body were used for *in vitro* fertilization (IVF) and SCNT.

### IVF

Thirty oocytes were transferred to 50 μL of BO-IVF medium (IVF Bioscience, Falmouth, UK) in one microdrop under mineral oil and 40 μL of sperm suspension (2–3 × 10^6^ sperm/mL) was added to the microdrop. IVF was done at 38.5°C, 5% CO_2_ for 10–12 h. The zygotes were transferred to a 25 mm concave glass dish, spermatozoa and cumulus cells were dispersed by gentle pipetting, and the zygotes were cultured in 400 μL of BO-IVC medium (IVF Bioscience, Falmouth, UK) with mineral oil covering the surface.

### SCNT

SCNT was performed according to a previous study (Liu *et al.* 2011). Goat fetal fibroblasts (GFFs) was isolated from a female fetus at Day 40 of gestation from a Saanen dairy goat. After removal of the head and internal organs of the female fetus, the remaining fetal tissue was minced with scissors for primary cell separation. Then GFFs were cultured to 2nd to 5th generations for nuclear transplantation. GFFs were maintained in Dulbecco’s modified Eagle’s medium (DMEM)/F-12 (Gibco) supplemented with 10% FBS (Gibco) at 37°C in a 5% CO_2_ incubator. GFFs were harvested by treatment with 0.025% trypsin + 0.5 mM ethylenediaminetetraacetic acid (EDTA) (Gibco) and starved by incubation in a medium containing 0.5% FBS (Gibco) the day before SCNT to ensure that all cells were arrested at G0 stage. The first polar body together with the metaphase plate were removed from the oocytes and synchronized GFFs with smooth shape were injected into the perivitelline space of the enucleated oocytes. The GFFs were fused with the oocytes by electrofusion exposure to two 20 ms, 1.2 kv/cm direct current (DC) pulses in fusion medium (BTXpress cytofusion medium C, Harvard Apparatus, Holliston, MA, USA). Lastly, reconstructed embryos were incubated for 2 h in TCM-199 and activated with 5 mM ionomycin for 5 min and 2 mM 6-DMAP for 4 h and then were cultured with BO-IVC.

### RNA sequencing (RNA-seq)

To identify the early embryonic transcripts and screen for differentially expressed lncRNAs during EGA of goat embryos, RNA-seq was conducted as described previously (Deng *et al.* 2019). In brief, three cDNA libraries were established from early goat embryos at two developmental stages, the two-cell (IVF-2c) and eight-cell embryos (IVF-8c and SCNT-8c). Each sample contained 15 embryos and one sample per stage and were transferred into lysis buffer for transcriptome sequencing at Novogene (Beijing, China). RNA-seq data were deposited in the Gene Expression Omnibus (GEO) at the National Center for Biotechnology Information (NCBI) under accession number GSE199933.

### lncRNA interference

To reduce the expression of *lncRNA3720*, a Smart Silencer system was formulated and synthesized by RiboBio (Guangzhou, China), comprised of six specific small interfering RNAs (siRNAs, 20 μM). The siRNA sequences are shown in Table 1. An unrelated RNA without any activity was provided as a negative control (NC, 20 μM). Using a Nikon inverted microscope with Eppendorf microinjector, we injected 5 pL of siRNAs (or NC RNA) into the cytoplasm after IVF. Eight-cell embryos were collected for reverse-transcription qPCR to validate interference efficiency, determine the target gene expression level and obtain statistics for the comparison of development rates.

**Table 1:** siRNA sequences for lncRNA interference.

siRNA ID	Target sequences
TCONS_00343720-SiRNA1	AGTTTGGAGTTGCCTTCAAC
TCONS_00343720-SiRNA2	GAGGATTCTCAGAGCTGCAG
TCONS_00343720-SiRNA3	AGAAGAAACTGAAGCACAA
TCONS_00343720-SiRNA4	GGTCTCTGACAGCACATAA
TCONS_00343720-SiRNA5	CTACCTCTTTGAAATGTAA
TCONS_00343720-SiRNA6	AGTTTGGAGTTGCCTTCAAC

### RNA isolation, reverse transcription, and real-time PCR

All primers were designed using Premier 5 based on sequencing data and synthesized by Tsingke (Beijing, China) (Table 2). Total RNA from samples (15 embryos/tube) was isolated by the Cells-to-Signal™ lysis buffer kit (Invitrogen, Austin, TX) according to the manufacturer’s instructions. The total RNA was reverse-transcribed into cDNA with the TransScript One-Step gDNA removal and cDNA synthesis SuperMix (TransGen Biotech). Real-time PCR was performed on an ABI StepOnePlus PCR system (Applied Biosystems) using ChamQ SYBR qPCR master mix (Vazyme). Each target gene was run three times, and the qPCR conditions were as follows: 95°C pre-denaturation for 30 seconds, followed by 40 cycles of denaturation at 95°C for 5 seconds, annealing and extension at 60°C for 30 seconds. The formula, *FC* = 2^−ΔΔCt^ was used to calculate relative gene expression.

**Table 2:** Primer pairs for qPCR.

Gene	Sequence (5′–3′)	
	Forward	Reverse
TCONS_00343720	GGGGAACAGAAGAAACTGAAGC	CTCCTGCTAAGACATTTGAGAACA
TCONS_00393472	CACCCTATCTCCATCCTCCTCT	AGCAGCCTCCGAAACAAATC
TCONS_00461730	ACCCGTGTCCTGTCTGATTG	CTCCGCAGAAAGAGAGAGGC
TCONS_00474517	GCACACTTCGCCAGCTAAAG	ATGGTCTGGCAAGATCGCTG
TCONS_00574621	GTCACTATGGGCCACCTCTT	GGATATGGCAACACCCACTG
TCONS_00562642	ACAGTCAGTGATGCTGGCTTTT	ATCCAACACCACCTTCCTACC
*ACTB*	CTGGGACGACATGGAGAAGATC	GCAGGGGTGTTGAAGGTCTC
*ZSCAN4*	AAAGCTACCTGACTTGGTCC	GTTCCCATGTTCATCCCTCCT
*EIF1AX*	CAAAGAAGATGGGCAAGAGTATG	GGTAGTCCCGTAGACCAACCA
*H2A* type 2-C	AAAAAGACGCGCATCATCCC	TTTGGCTTTATGGCTTTCGGT
*H2B* type 1	GCCGTAAAGAAAGTTATTCCGTG	TATAATGAGCCAAGCGCGATG
*IPMK*	GGGCACATGTACGGGAAAGA	ACAATCAGCAGCAAAAACCAT
*RRM2*	ATGCCATTGAGACGATGCCT	AGCAAAGGCTACCACACGTT
*TM4SF1*	GCTGGAACAGGAAGACTGCT	TGGCATCCATGCATCTTGGT
*UBE2C*	CTTCCCTGAATCGGACAACCT	CTTCACCGTGGGTGCGTT
*MT2A*	AAAGATTGCAAGTGCGCCTC	AGCAACTGCACTTGTCCGAG
*lncRNA3720*	TAACTGCTCGGCGTCGTCAT	CAACTCCAAACTCACTGTCACATCC
*U6*	CGCTTCGGCAGCACATATAC	TTCACGAATTTGCGTGTCAT
*GAPDH*	CGACTTCAACAGCGACACTCAC	CCCTGTTGCTGTAGCCCAATTC

### Rapid amplification of cDNA ends (RACE)

Total RNA was isolated from goat tissue using TransZol (TransGen Biotech) and reverse-transcribed into 5’- and 3’-RACE-Ready cDNA. The 5’-full RACE and 3’-full RACE were performed using a SMARTer® RACE 5’/3’ kit (Takara, Dalian, China) according to the manufacturer’s protocol. The reaction products were identified by electrophoresis on agarose gels.

### Construction of plasmid vectors

The *lncRNA3720* gene-specific primers that incorporated 5’*Hind* III and 3’*Xhol* I restriction sites were used to amplify *lncRNA3720* cDNA derived from reverse-transcribed RNA. The PCR product was cloned into the plasmid vector pcDNA3.1(+) after the CMV promoter to generate pcDNA3.1(+)–lncRNA3720.

### Cell culture and transfection

GFFs were maintained in DMEM)/-12 (Gibco) supplemented with 10% FBS (Gibco) at 37°C in a 5% CO_2_ incubator. Cells were harvested by trypsin/ethylenediaminetetraacetic acid (EDTA) (0.025% trypsin, 0.5 mM EDTA) (Gibco). GFFs were transfected with pcDNA3.1(+-*lncRNA3720* using Lipofectamine 3000 (Invitrogen). After transfection (36 h), the cells were harvested, total RNA was extracted using TRIzol reagent and reverse transcription was carried out with TransScript one-step gDNA removal and cDNA synthesis SuperMix, according to the manufacturer’s protocol. The efficiency of transfection was determined by quantitative real-time PCR (qRT-PCR).

### Subcellular localization of *lncRNA3720*

The subcellular localization and function of *lncRNA3720* in GFFs was determined by transient expression of the pcDNA3.1(+)-*lncRNA3720* fusion protein and utilization of a nuclear and cytoplasmic protein extraction kit (Beyotime Biotechnology, Shanghai, China).

### *In vitro* transcription

The *lncRNA3720* cDNA was amplified by PCR with primers linked to the T7 promoter, to generate a template for *in vitro* transcription. The* in vitro* transcription products were recovered using an mMESSAGE mMACHINE T7 kit (Invitrogen, Thermo Fisher Scientific) and purified by LiCl precipitation.

### Immunofluorescence (IF)

IF staining was performed according to a previous study (Su *et al.* 2011). Samples were fixed with 4% paraformaldehyde and rinsed three times, 5 min each, with PBS-PVA (PBS containing 0.2% PVA) and permeabilized with 0.1% Triton X-100 for 30 min at room temperature. Samples were incubated overnight with anti-H2A type 2-C (1:1000; ab45152, Abcam), anti-H2B type 1 (1:1000; ab177430, Abcam) or anti-CDX2 (1:100; EPR2764Y, Invitrogen) at 4°C. Afterward, the samples were rinsed three times for 5 min each with PBS-PVA, incubated with goat anti-rabbit IgG H&L (Alexa Fluor® 488) (1:500; ab150077, Abcam) or goat anti-mouse IgG (H+L) highly cross-adsorbed secondary antibody, Alexa Fluor™ Plus 555 (1:500; A32727, Invitrogen) for 2 h at room temperature in the dark and rinsed with PBS-PVA. DAPI (1:500; C1005, Beyotime) nuclear stain was added for 8 min at room temperature in the dark, and rinsed three times with PBS-PVA. The specific signals were observed and imaged using a laser scanning confocal microscope (Carl Zeiss, Germany) under the same conditions. Fluorescence signal intensities were measured according to a previous report (Shi *et al.* 2023).

### Western blot

Western blotting was carried out following a previous study from our laboratory (Han *et al.* 2018). GFFs transfected with pcDNA3.1(+)-*lncRNA3720* were transferred into lysis buffer and total protein was extracted. Protein aliquots were separated by SDS–PAGE on a 5% stacking gel, and a 10% separating gel at 120 V for 1.5 h and then electrophoretically transferred onto a polyvinylidene fluoride membrane at 250 mA for 2.5 h. Membranes were blocked in TBST (Tris-buffered saline pH 7.4 with 0.1% Tween 20) containing 3% BSA for 2 h at room temperature and then incubated overnight with anti-H2A type 2-C (1:1000; ab45152, Abcam) or anti-H2B type 1 (1:500; ab177430, Abcam) or anti-β-actin (1:500; HC201-01, Trans) at 4°C. After washing three times with TBST for 10 min each time, the membrane was incubated for 2 h at room temperature with horseradish peroxidase-labeled goat anti-rabbit IgG(H+L) (1:1000; A0208, Beyotime). After washing three times with TBST, 10 min each time, the signals were detected using ECL (Beyotime). The intensity values of the different protein bands were measured by Image J (Media Cybernetics, Silver Spring).

### Statistical analysis

The statistical analysis was performed using GraphPad Prism 6.0 (GraphPad Software). Data are presented as mean ± standard deviations. The Tukey–Kramer test for one-way ANOVA was used to analyze data with three or more groups, and Student’s two-tailed *t*-test was used to analyze comparisons with two groups. Values of* P* < 0.05 was considered statistically significant.

## Results

### Screening of differentially expressed lncRNAs from transcriptome of IVF and SCNT embryos

**Figure 1:**
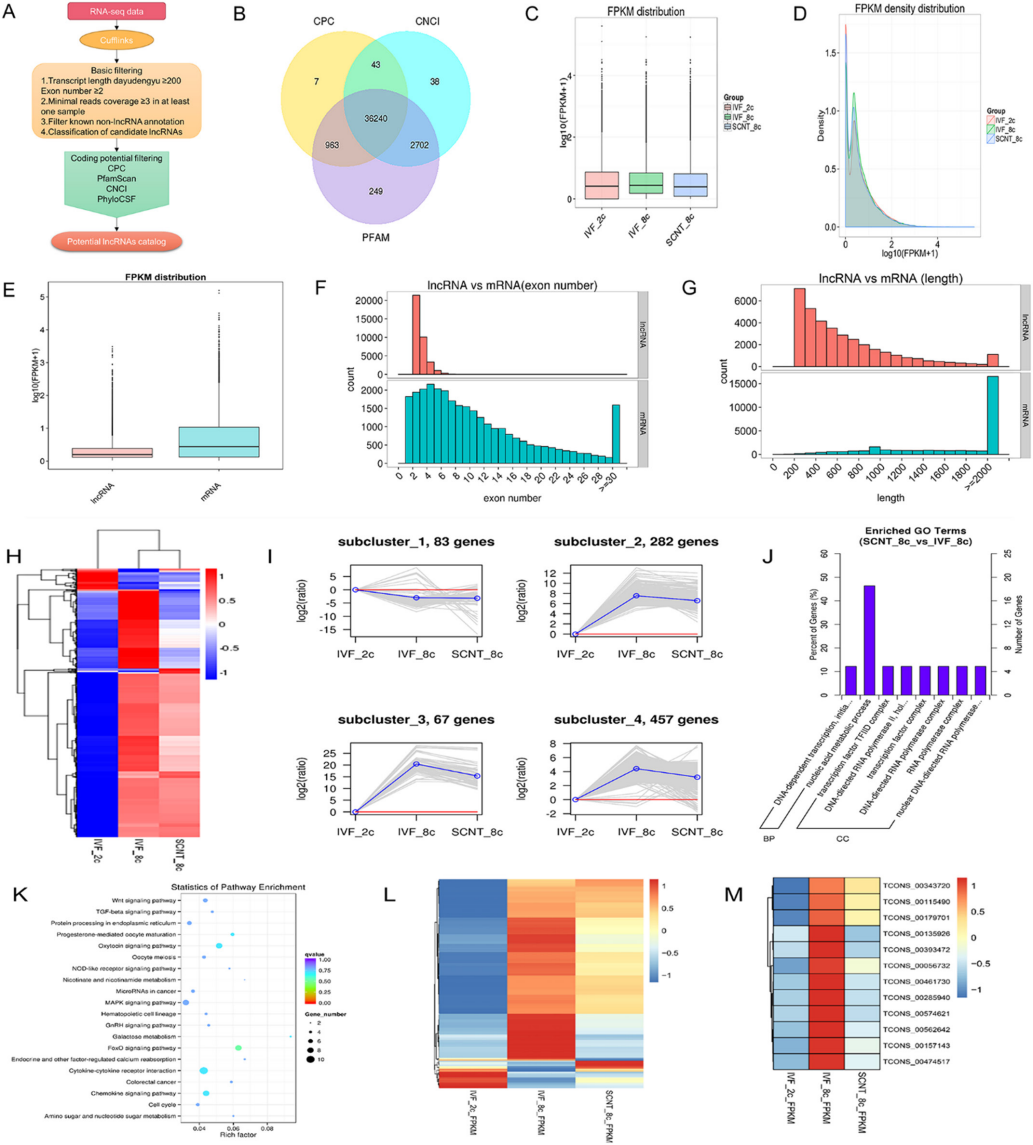
Screening of lncRNA expression in IVF and SCNT embryos of goat and identification of differentially expressed lncRNAs. (A) Workflow for analysis of lncRNA libraries. (B) Venn diagram with the numbers in each large circle representing the total number of noncoding transcripts predicted by the software, and the overlapping parts of the circles representing the number of noncoding transcripts in common predicted by the software. (C, D) The FPKM distribution of DEGs in IVF-2c, IVF-8c and SCNT-8c. (E) The FPKM distribution of differentiallyexpressed mRNAs and lncRNAs. (F) The exon numbers of differentially expressed mRNAs and lncRNAs. (G) The length of differentially expressed mRNAs and lncRNAs. (H) Heatmap showing normalized expression patterns (FPKM) of DEGs. Red: relatively high expression. Blue: relatively low expression. (I) Four clusters of differentially expressed lncRNAs and mRNAs. (J) GO analysis of DEGs. (K) KEGG analysis of DEGs. (L) Heatmap showing differentially expressed lncRNAs identified by pairwise comparison between two- and eight-cell IVF and SCNT embryos. (M) The 12 candidate lncRNAs differentially expressed in the three groups.

To identify functional lncRNAs during EGA in goat early embryos, we analyzed the transcriptomes of IVF-2c, IVF-8c and SCNT-8c embryos by RNA-seq. Illumina's HiSeq platform was used to sequence the libraries constructed by goat IVF-2c, IVF-8c and SCNT-8c. According to the workflow in Fig. 1A and the summarized results of coding potential calculator (CPC), coding-non-coding-index (CNCI) and protein family database (PFAM), a total of 36,240 lncRNAs was obtained from the three groups (Fig. 1B). On this basis, the expression level was further screened, and the pair-to-pair comparison between IVF-8c and IVF-2C and IVF-8c and SCNT-8C was performed according to the FPKM (fragments per kilobase of exon model per million mapped fragments) value (Fig. 1C, D and E), and the corrected *q* value was calculated respectively. The length distribution and the number of exons of screened lncRNAs were determined (Fig. 1F and G). After selections for *q* < 0.05 and | Log2 Fold change| >2, 889 mRNAs and lncRNAs were found to be differentially expressed among the three groups (Fig. 1H) and were further subdivided into four clusters (Fig. 1I). There were 61 lncRNAs and 50 mRNAs highly expressed in IVF-8c embryos compared to SCNT-8c embryos. Results of gene ontology (GO) showed that the genes with significant differences were mainly related to nucleic acid metabolism pathways (Fig. 1J). The Kyoto Encyclopedia of Genes and Genomes (KEGG) enrichment analysis revealed that the differentially expressed genes were enriched in oocyte meiosis and Wnt-signaling pathway (Fig. 1K). We found 194 differentially expressed lncRNAs between IVF-8c and IVF-2c and SCNT-8c (Fig. 1L). And in those genes, there were only 12 differentially expressed lncRNAs that were highly expressed in IVF-8c but decreased in IVF-2c and SCNT-8c. Lastly, we screened 12 candidate lncRNAs for potential influence on early embryonic development (Fig. 1M).

### Identification of key lncRNAs that affect early embryonic development

**Figure 2:**
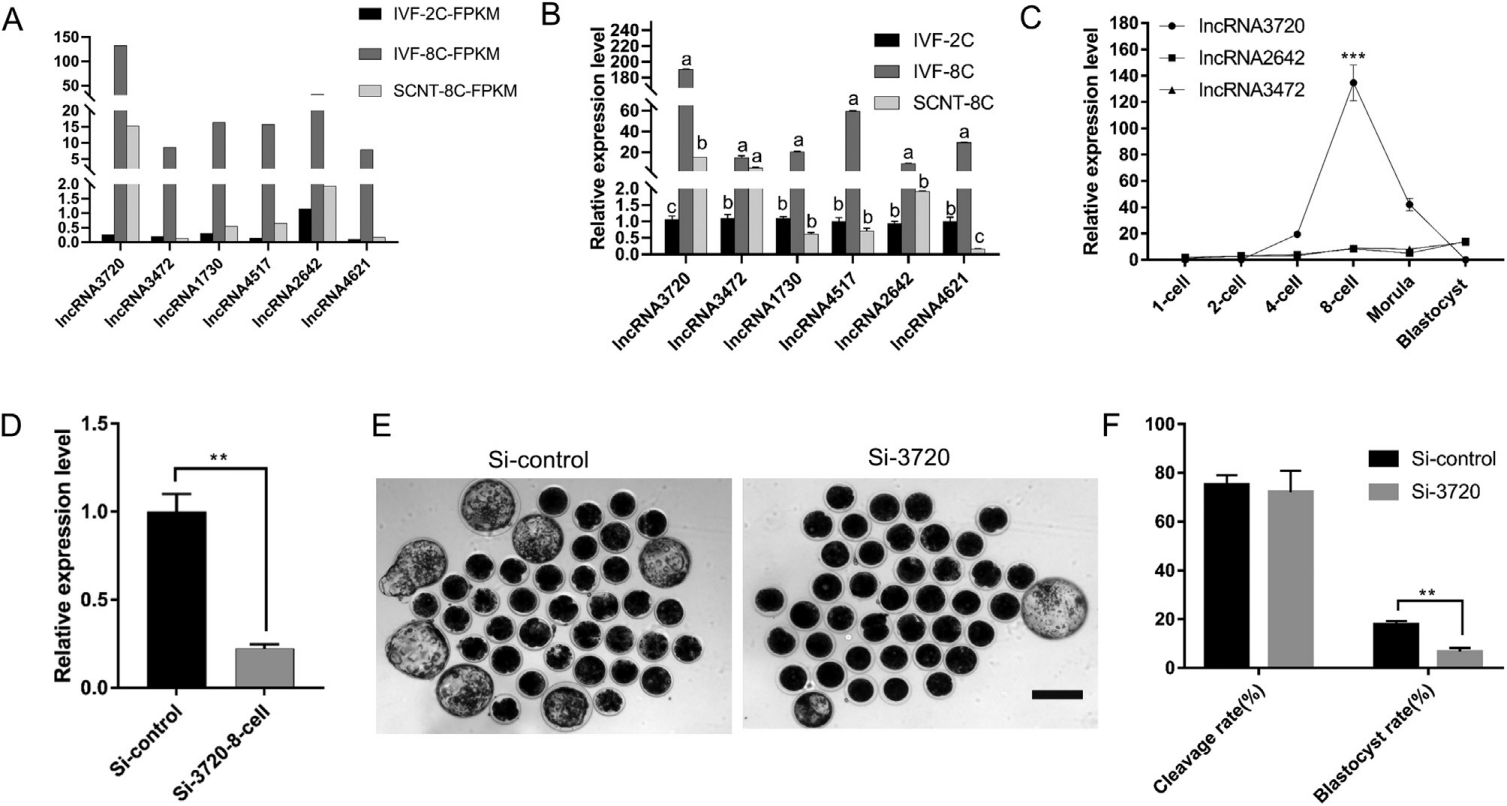
Expression profiles of lncRNAs during EGA. (A) Analysis of overall transcript levels of the selected lncRNAs. (B) Expression of selected lncRNAs by qRT-PCR with β-actin used as control reference gene. Different lowercase letters (a, b, c) above plots indicate significant differences (*P* < 0.05). Forty-five embryos were used for each gene. (C) qRT-PCR results showing expression levels of key lncRNAs in goat embryos at different developmental stages. ***P < 0.01. Thirty embryos were used per stage. (D) Validation of the efficiency of Smart Silencer-mediated *lncRNA3720* knockdown in eight-cell-stage embryos using qRT-PCR. **P < 0.01. Forty and 41 embryos were used for the Si-control group and the Si-3720 group, respectively. (E) Representative images of Si-control embryos and Si-3720 embryos at day 8. Scale bar, 100 µm. (F) Effect of lncRNA3720 knockdown on embryo cleavage rate and the development of embryos to the blastocyst stage. Analyses of cleavage rate and blastocyst rate in Si-control and Si-3720 group* in vitro*. **P< 0.01. Forty-two embryos were used in each group.

In order to test the accuracy of RNA-seq, we randomly selected 6 of the 12 candidate lncRNAs for qPCR assay and found that the results were consistent with the sequencing. The number of FPKM fragments for the six lncRNAs was calculated by Cuffdiff (v2.1.1) (Fig. 2A). The expression levels of the six lncRNAs were verified by RT-qPCR, and the results were in accord with sequencing data (Fig. 2B). Some studies have shown that lncRNAs and their target genes are usually transcribed at the same site or in close proximity and may regulate the expression of adjacent protein-coding genes in a positive or negative manner (Tsai *et al.* 2010, Yin *et al.* 2015, Liu *et al.* 2019*a*). Therefore, we searched for protein-coding genes within 10 kb upstream or downstream of the candidate lncRNAs as potential target genes associated with embryonic development. Then we selected three key lncRNAs-*lncRNA3720*, *lncRNA3472* and *lncRNA2642* from the 12 candidate lncRNAs according to the correlation between the upstream- and downstream-predicted target genes (Pang & Thomas 2010, Neutzner *et al.* 2011, Chang *et al.* 2012). Subsequently, we analyzed the expression levels of *lncRNA3720*, *lncRNA3472* and *lncRNA2642* during early embryonic development. The results showed that *lncRNA3720* was highly expressed in eight-cell-stage embryos (Fig. 2C). The results were consistent with the hypothesis that lncRNA3720 was an active part of the EGA process; therefore, we selected *lncRNA3720* to investigate for its effects on target genes and how this might regulate the development of early goat embryos.

To test the effect of *lncRNA3720* on the early embryonic development of goats, we knocked down the *lncRNA3720* expression level by injecting antisense oligonucleotides and siRNAs into the cytoplasm of zygotes. We verified the interference efficiency at the eight-cell stage by using RT-qPCR. The interference efficiency was approximately 85% by injecting Smart Silencer into the cytoplasm of zygotes compared with the control (Si-control) (Fig. 2D). Knockdown of *lncRNA3720* caused a developmental block at the eight-cell stage in goat embryos, and the blastocyst rate was significantly lower than that in the control group (Fig. 2E and F), showing that the expression level of *lncRNA3720* affected early embryonic development in goats.

### *lncRNA3720* elevated the expression of key EGA genes

**Figure 3:**
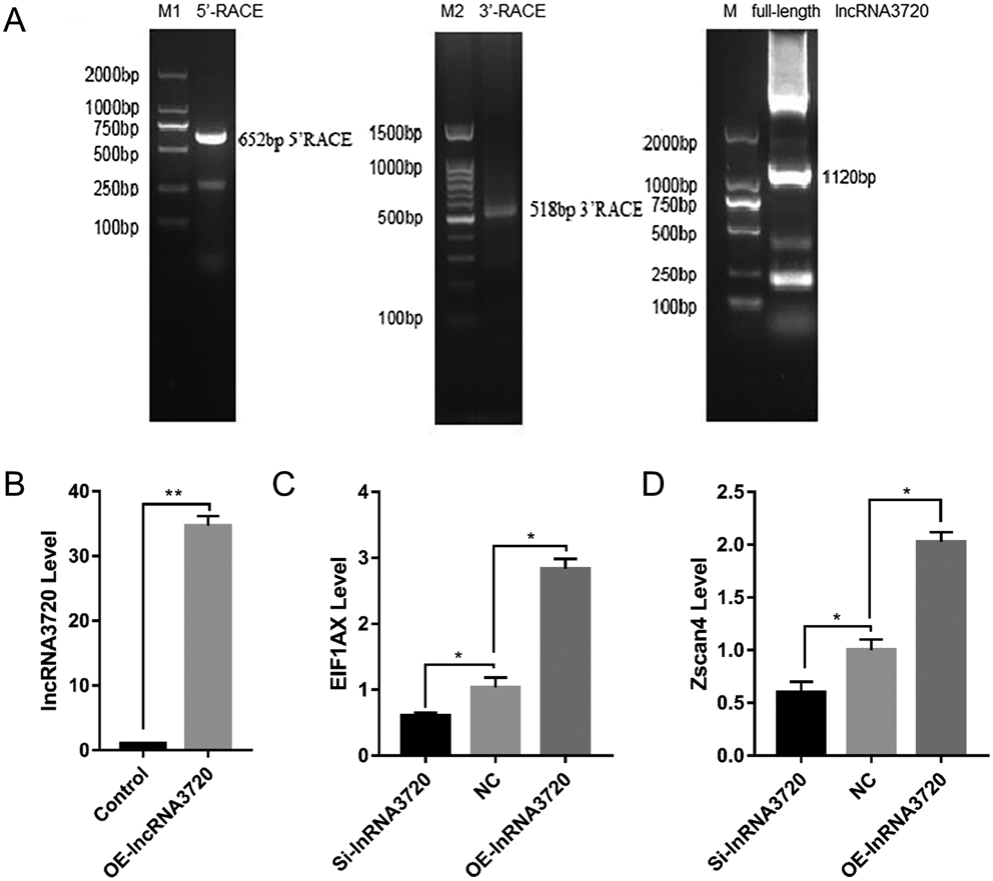
Effect of *lncRNA3720* on EGA genes. (A) The full-length sequence of *lncRNA3720* was amplified by RACE. M1, Trans2K® DNA marker; M2, 100 bp DNA ladder; M, Trans2K® DNA marker. (B) Verification of overexpression efficiency of *in vitro*-transcribed *lncRNA3720* in IVF eight-cell-stage embryos. OE (*in vitro*-transcribed *lncRNA3720* injection). **P < 0.01. Thirty embryos were used in each group. (C, D) Analysis of EGA genes in IVF eight-cell-stage embryos after knockdown or overexpression of *lncRNA3720*. Si, *lncRNA3720* Smart Silencer injection; OE, *in vitro*-transcribed lncRNA3720 injection; NC, non-target control RNA injection. *P < 0.05. Thirty embryos were used in each group.

EGA is necessary for embryonic development and the process can be divided into major EGA and minor EGA stages. The timing of major EGA is related to the species. Goat major EGA involves expression of a large number of embryonic genes and occurs at the eight-cell stage. We hypothesized that the developmental arrest of eight-cell-stage embryos caused by *lncRNA3720* knockdown may be related to abnormal EGA initiation, so we determined the expression of the EGA initiation genes, *ZSCAN4* and* EIF1AX,* by RT-qPCR. In order to obtain the full-length sequence of l*ncRNA3720*, we performed 5’-RACE and 3’-RACE to amplify the end sequences of *lncRNA3720* cDNA and obtained the full-length (1120 bp) sequence of *lncRNA3720* (Fig. 3A). The sequence was confirmed as *lncRNA3720* by sequencing and used for* in vitro* transcription (Fig. 3B). *In vitro*-transcribed *lncRNA3720* and Smart Silencer were microinjected into IVF embryos. The expression of *ZSCAN4* and *EIF1AX* in embryos was decreased by *lncRNA3720* knockdown, and increased by *lncRNA3720* overexpression (Fig. 3C and D). Thus, *lncRNA3720* could upregulate expression of the key EGA genes, *ZSCAN4* and *EIF1AX*.

### Determination of the potential target genes of *lncRNA3720*

**Figure 4:**
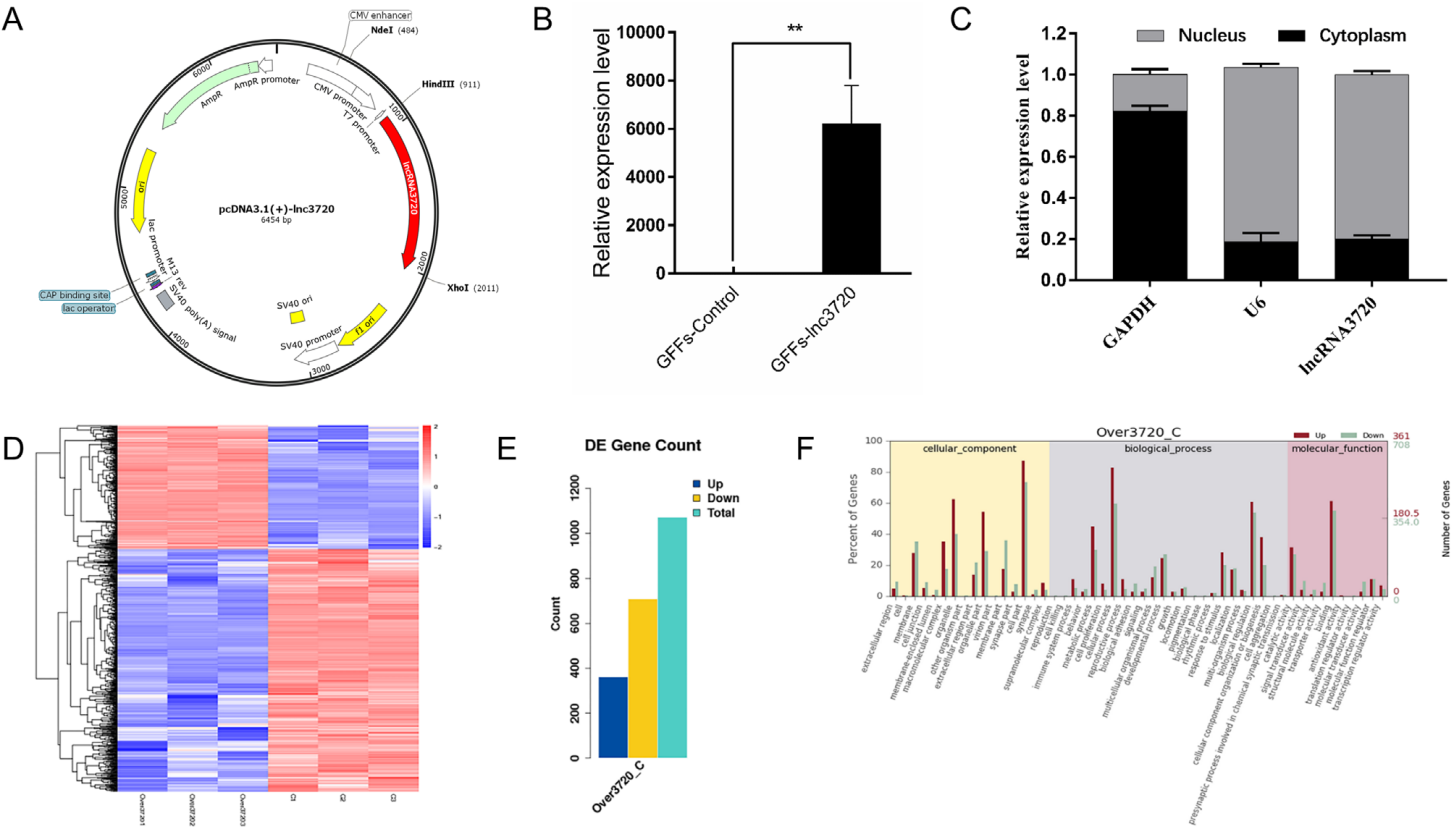
Analysis of potential target genes of *lncRNA3720* from the transcriptome of GFFs and embryos. (A) Plasmid construct of goat pcDNA3.1(+)-*lncRNA3720* eukaryotic expression vector. The red indicates the insertion position of *lncRNA3720*. (B) Transfection efficiency of the *lncRNA3720* plasmid transfection group compared with the control group. ^**^ *P* < 0.01. (C) Subcellular localization of l*ncRNA3720*. (D) Heatmap showing the degree of difference between DEGs from controls and GFFs overexpressing *lncRNA3720*, with red representing relatively high expression and blue representing relatively low expression. (E) Histogram showing number of DEGs, with blue representing upregulation, yellow downregulation and green the total number of DEGs. (F) Gene ontology (GO) enrichment of DEGs between the control and overexpression groups.

**Figure 5:**
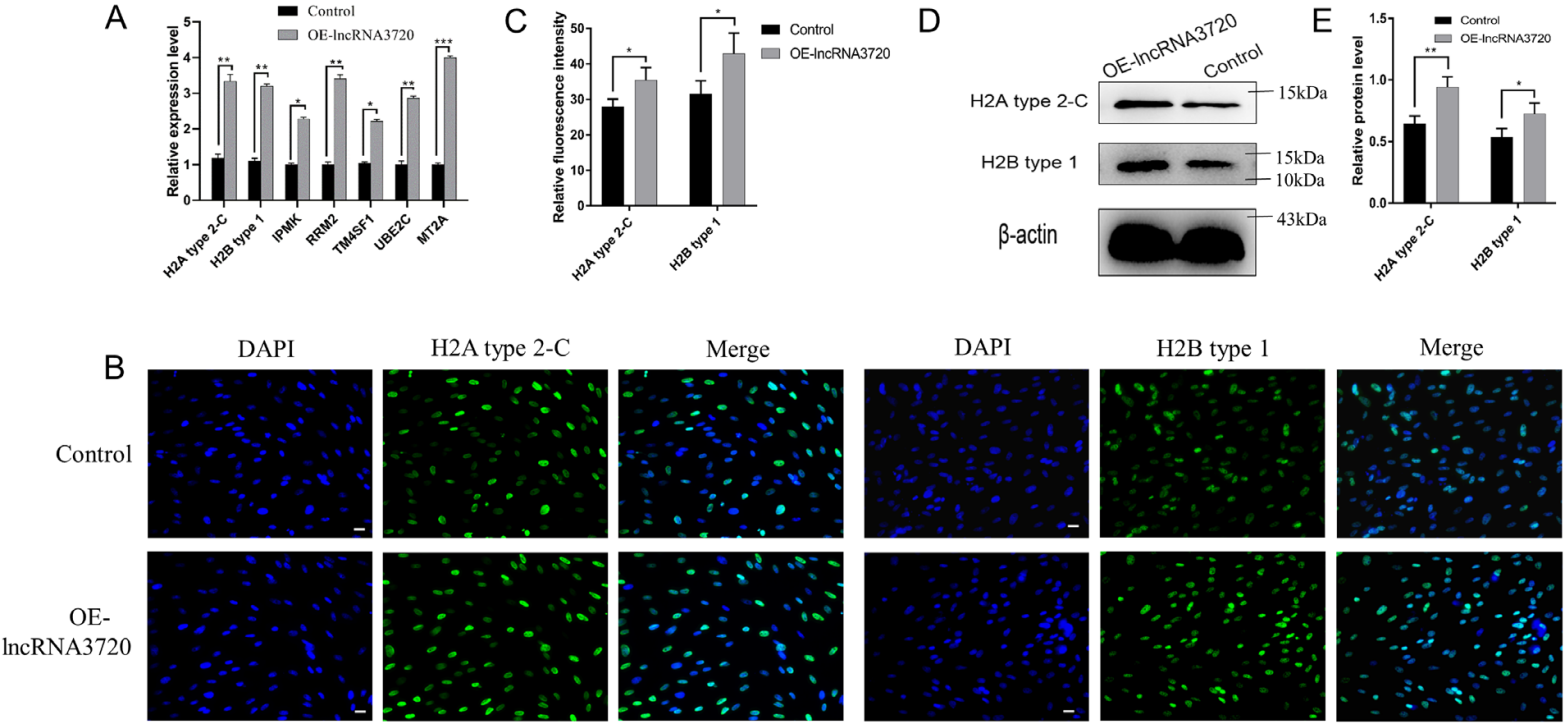
Regulation of histone H2A variant expression by *lncRNA3720*. (A) Expression of the seven genes from GFFs overexpressing *lncRNA3720*. OE (the *lncRNA3720* plasmid transfection group). ^*^P < 0.05,^ **^P < 0.01, ^***^P < 0.01. (B) Immunofluorescence staining for histone H2A type 2-C and H2B type 1. OE (the *lncRNA3720* plasmid transfection group). Scale bar, 20 µm. (C) Fluorescence intensity of histone *H2A* type 2-C and *H2B* type 1 in GFFs. ^*^P < 0.05. (D) Western blotting of histone *H2A* type 2-C and *H2B* type 1 in GFFs. (E) Relative protein level of histone H2A type 2-C and H2B type 1. ^*^P < 0.05,^ **^P < 0.01.

**Figure 6:**
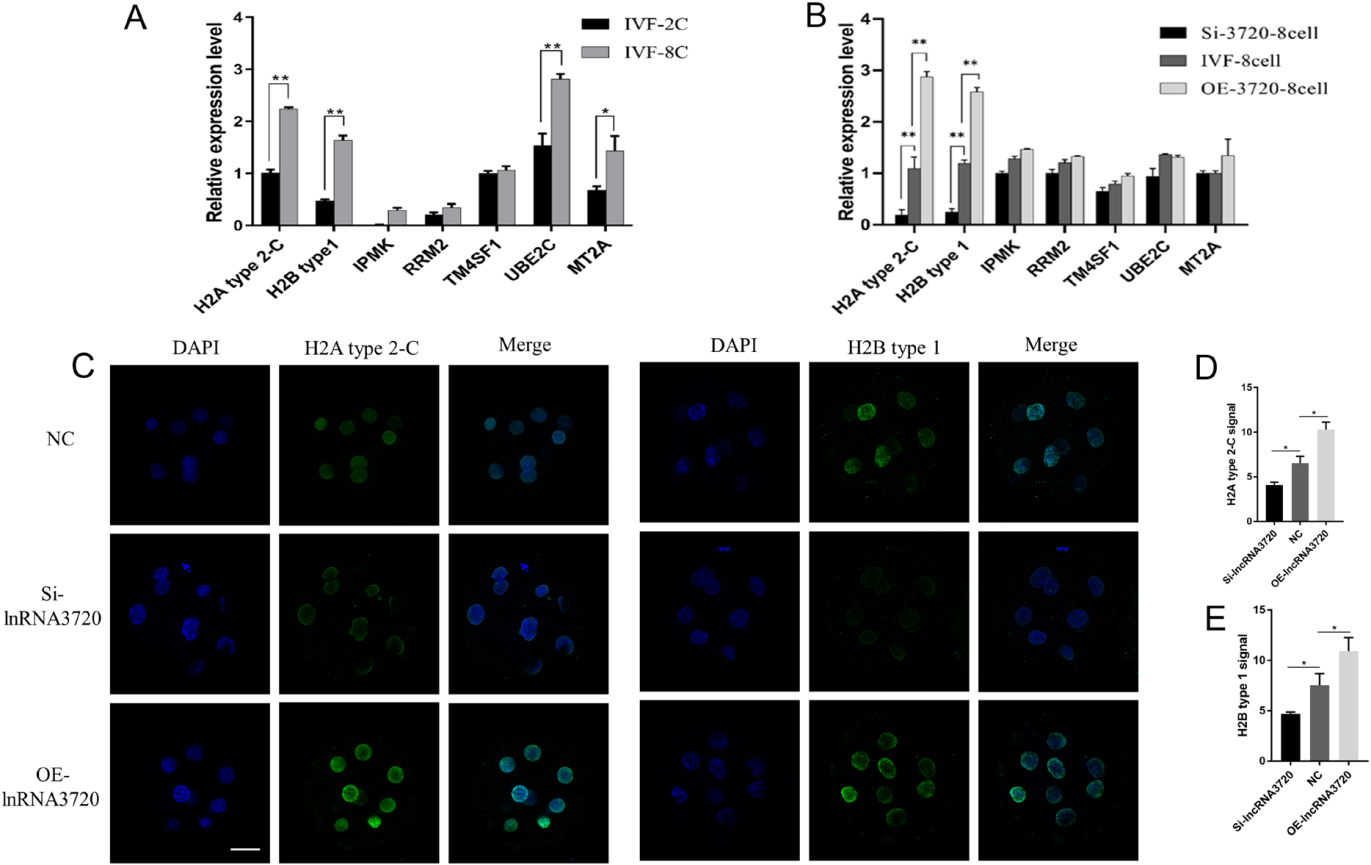
*lncRNA3720* maintains embryonic development by regulating histone H2A variant expression. (A) Analysis of overall transcript levels of the seven potential target genes in IVF-2c and IVF-8c embryos. ^*^P < 0.05, ^**^P < 0.01. Thirty embryos were used per gene. (B) Expression of the seven genes in IVF eight-cell-stage embryos after knockdown and overexpression of *lncRNA3720*. Si,* lncRNA3720* Smart Silencer injection; OE, *in vitro*-transcribed *lncRNA3720* injection. ^**^P < 0.01. Forty-five embryos were used per gene. (C) Immunofluorescence staining for histone *H2A* type 2-C and *H2B* type 1 expression. Scale bar, 50 µm. NC, non-target control RNA injection; Si, *lncRNA3720* Smart Silencer injection; OE, *in vitro*-transcribed *lncRNA3720* injection. (D) Fluorescence intensity of histone *H2A* type 2-C. ^*^P < 0.05. (E) Fluorescence intensity of histone *H2B* type 1. ^*^P < 0.05.

To identify the genes regulated by *lncRNA3720* in GFFs, we first constructed a eukaryotic expression vector, pcDNA3.1(+)-*lncRNA3720*, through the *Hind* III and *Xhol* I restriction sites (Fig. 4A). RT-qPCR results showed that *lncRNA3720* could be overexpressed in GFFs after transient transfection (Fig. 4B). Meanwhile, *lncRNA3720* localized mainly in the nucleus by means of nucleoplasmic isolation and RT-qPCR (Fig. 4C). To identify the possible target genes of *lncRNA3720*, we performed transcriptome sequencing to compare gene expression between pcDNA3.1(+) and pcDNA3.1(+)-l*ncRNA3720* overexpressed in GFFs. We defined a DEG as having FDR <0.05 and FC >2. The clustering of transcriptional differences between groups was shown by heat map analysis (Fig. 4D). A total of 1071 DEGs was obtained, among which 362 genes were upregulated and 709 genes were downregulated (Fig. 4E). The GO enrichment analysis suggested that DEGs were mainly enriched in cell processes, biological regulation and molecular functions such as molecular binding and catalytic activity (Fig. 4F). Next, we compared the DEGs between pcDNA3.1(+) and pcDNA3.1(+)-*lncRNA3720* GFFs and between IVF-2c and IVF-8c embryos. In both IVF-8c embryos and *lncRNA3720*-overexpressed GFFs, we found that seven genes were upregulated. The quantitative verification result showed that histone *H2A* type 2-C and histone *H2B* type 1 were significantly upregulated in GFFs overexpressing *lncRNA3720* (Fig. 5A). The IF and western blot results also indicated that the signal intensity of histone* H2A* type 2-C and histone *H2B* type 1 was significantly increased (Fig. 5B, C, D and E). The expression levels of the seven potential target genes in IVF-2c and IVF-8c embryos were verified by RT-qPCR (Fig. 6A). After knockdown or overexpression of *lncRNA3720* in IVF embryos, we found that *lncRNA3720* mainly regulated the expression of histone *H2A* type 2-C and *H2B* type 1 as important target genes of *lncRNA3720* (Fig. 6B). The IF results showed that the signal intensity of histone* H2A* type 2-C and histone *H2B* type 1 was significantly increased after overexpression of *lncRNA3720* and decreased after the knockdown of *lncRNA3720* (Fig. 6C, D and E).

### Overexpression of *lncRNA3720* improves the developmental potential of SCNT embryos

**Figure 7:**
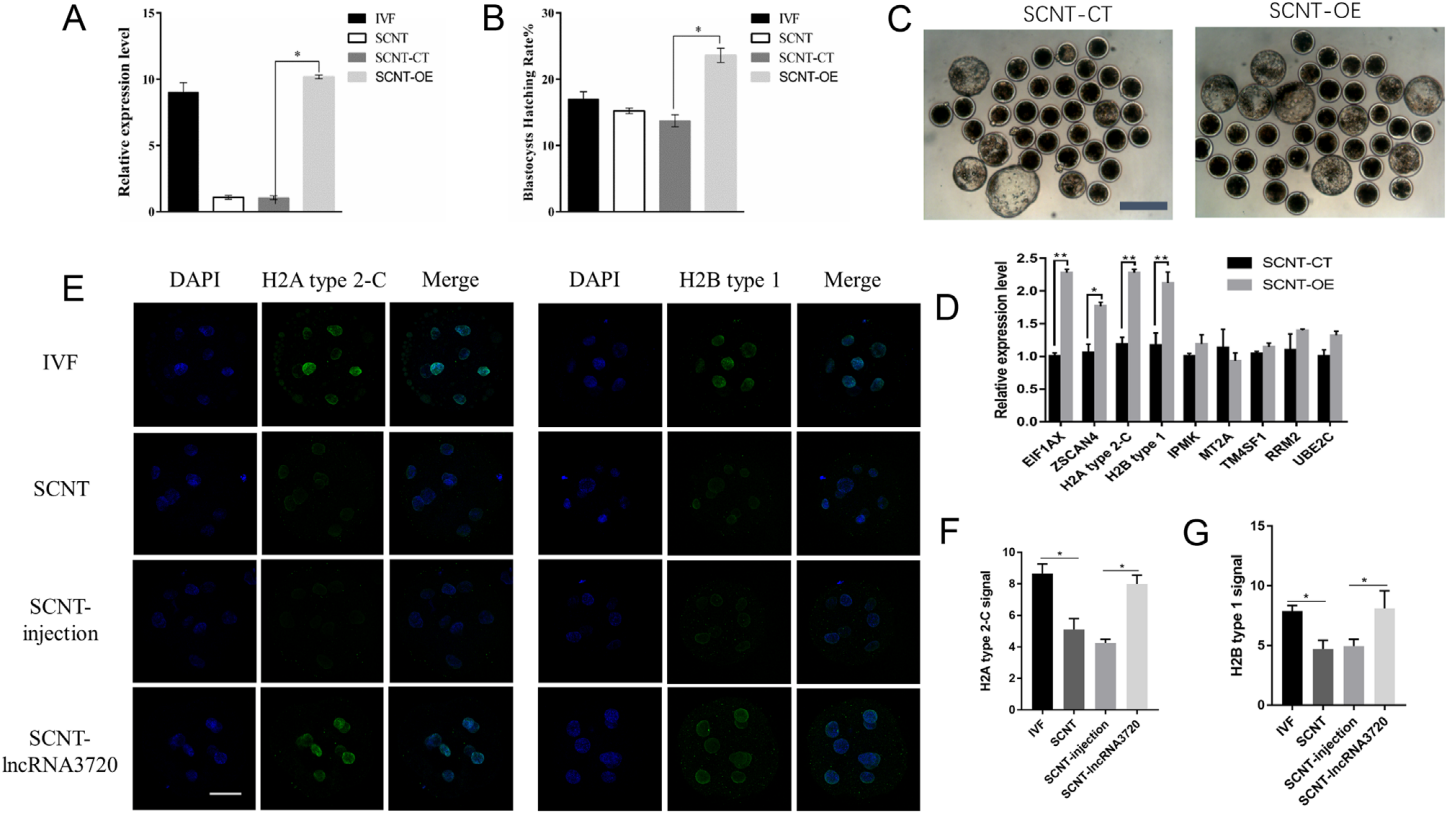
Nuclear transfer embryo compensation experiment. (A) Expression of *lncRNA3720* in IVF embryos, SCNT embryos, SCNT-CT embryos and SCNT-OE embryos by Q-PCR. SCNT-CT, non-target control RNA injection; SCNT-OE, *in vitro*-transcribed *lncRNA3720* injection. ^*^P < 0.05. Twenty embryos were used in each group. (B) Blastocyst rate of IVF embryos, SCNT embryos, SCNT-control embryos and SCNT-*lncRNA3720* embryos. SCNT-CT, non-target control RNA injection, SCNT-OE (*in vitro*-transcribed *lncRNA3720* injection). ^*^P < 0.05. Thirty, 35, 37 and 40embryos were used for the IVF, SCNT, SCNT Control and SCNT-OE groups, respectively. (C) Representative images of SCNT-control embryos and SCNT-*lncRNA3720* embryos at 8 days. Scale bar, 100 µm. (D) Potential target gene expression after nuclear transfer embryo compensation. SCNT-CT (non-target control RNA injection), SCNT-OE (*in vitro*-transcribed *lncRNA3720* injection). ^*^P < 0.05, ^**^P < 0.01. Thirty embryos were used for one gene. (E) Immunofluorescent staining of histone H2A type 2-C and histone H2B type 1 expression in eight-cell-stage embryos. SCNT-injection (non-target control RNA injection), SCNT-*lncRNA3720* (*in vitro*-transcribed *lncRNA3720* injection). Scale bar, 50 µm. (F) Fluorescence intensity of histone H2A type 2-C. SCNT-injection (non-target control RNA injection), SCNT-*lncRNA3720* (*in vitro*-transcribed *lncRNA3720* injection). ^*^P < 0.05. (G) Fluorescence intensity of histone H2B type 1. SCNT-injection (non-target control RNA injection), SCNT–*lncRNA3720* (*in vitro*-transcribed *lncRNA3720* injection). ^*^P < 0.05.

Our results showed that the expression level of *lncRNA3720* influenced early development of IVF embryos and that the expression of *lncRNA3720* was lower in SCNT embryos; thus, we hypothesized that overexpression of *lncRNA3720* might improve the developmental potential of SCNT embryos. We injected* in vitro*-transcribed *lncRNA3720* into SCNT embryos to correct the low level of *lncRNA3720* (Fig. 7A) and showed that the blastocyst rate of SCNT embryos was increased (Fig. 7B and C). The expression of *EIF1AX*, *ZSCAN4*, *H2A* type 2-C and histone *H2B* type 1 were significantly increased by overexpressing *lncRNA3720* (Fig. 7D). The immunofluorescence indicated that the signal intensity of *H2A* type 2-C and* H2B* type 1 was significantly increased (Fig. 7E, F and G).

**Figure 8:**
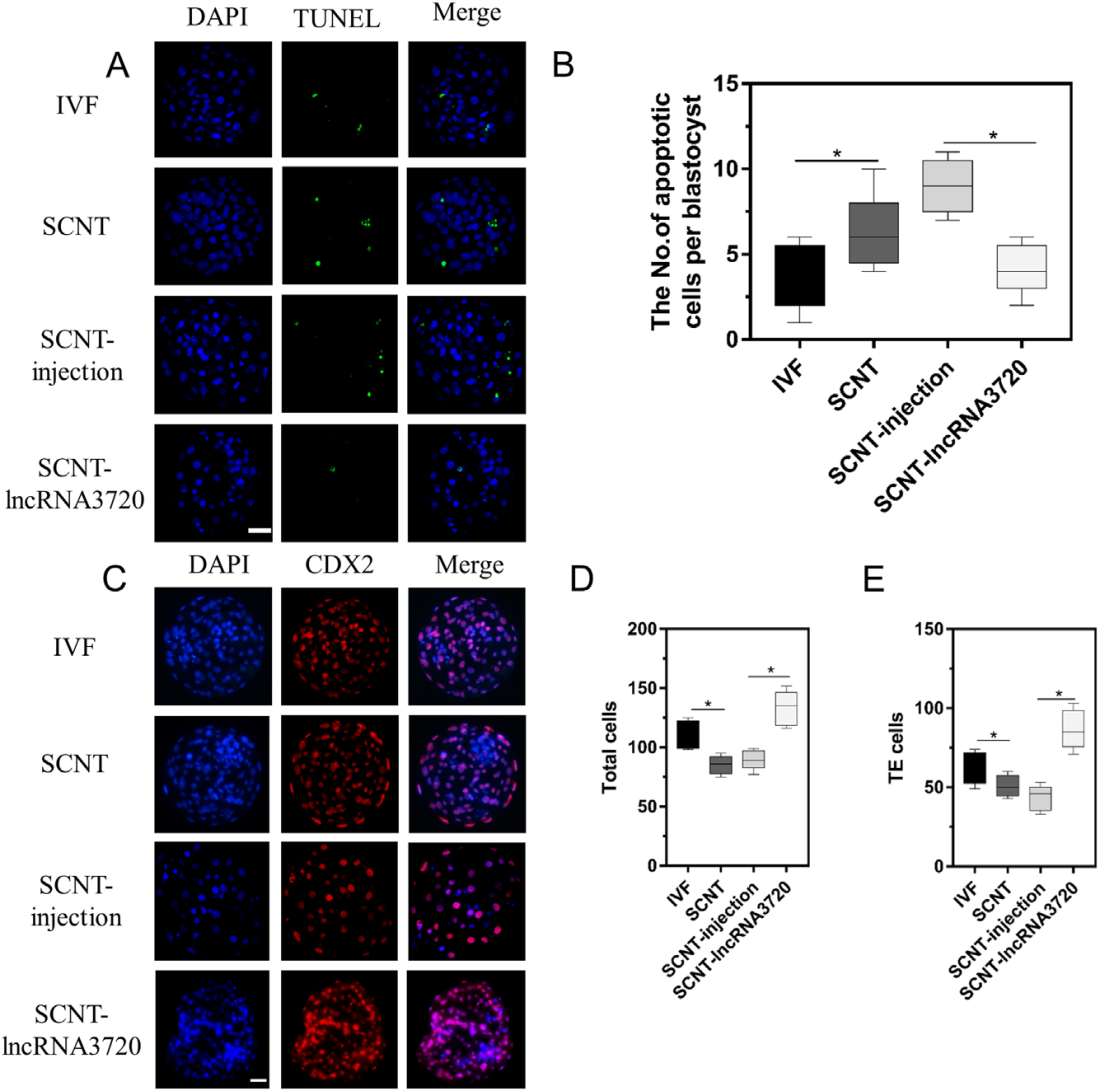
Nuclear transfer embryo compensation affects blastocyst quality. (A) The apoptotic cells show a green fluorescent signal in blastocysts. SCNT-injection (non-target control RNA injection), SCNT–*lncRNA3720* (*in vitro*-transcribed *lncRNA3720* injection). Scale bar, 50 μm. (B) Box plots indicate the number of apoptotic cells per blastocyst. *P < 0.05. Ten blastocysts were used in each group. (C) The TE and ICM nuclei were stained with DAPI (blue), the CDX2 antibody was indirectly labeled with Texas red resulting in a red fluorescent signal in TE cells in blastocysts. Scale bar, 50 μm. (D) Box plots show total cell numbers in blastocysts. *P < 0.05. Ten blastocysts were used in each group. (E) Box plots show the number of TE cells in blastocysts. *P < 0.05. Ten blastocysts were used in each group.

Embryo morphology assessment is the most commonly used parameter for measuring embryo quality. The segregation between the trophectoderm (TE) cells and the inner cell mass (ICM) occurs at the morula stage and continues during the blastocyst stage. Poor quality embryos may exhibit various features of apoptosis. Therefore, numbers of total cells, the ratio of the inner cell mass to the trophectoderm (ICM/TE) and the percent of apoptotic cells provide key information for the evaluation of embryo quality (Wydooghe *et al.* 2011). According to the terminal deoxynucleotidyl transferase dUTP nick-end labeling (TUNEL) assay, calculation of the percentage of apoptotic cells in blastocysts (Fig. 8A) showed that the level of apoptosis in the presence of overexpressed *lncRNA3720* was significantly lower than that in SCNT-injection embryos (Fig. 8B). Total and TE cell numbers were counted after CDX2 staining (Fig. 8C) and showed that the number of total and TE cells with overexpressed *lncRNA3720* was significantly higher than in SCNT-injection embryos (Fig. 8D and E).

## Discussion

In recent years, an increasing number of studies have found that abnormal epigenetic modification is an important factor affecting the development of SCNT embryos. The efficiency of SCNT in mice (Liu *et al.* 2016*a*), cattle (Liu *et al.* 2018) and goats (Han *et al.* 2018) can be significantly improved by treating donor cells or embryos with epigenetic regulatory factors. This study is the first to show that overexpression of *lncRNA3720* significantly improves the development rate and quality of SCNT embryos and confirms the importance of assessing the function of non-coding sequences for understanding the unique early embryonic development mechanism of domestic animals. SCNT is a complex process, which can reprogram terminally differentiated cells into a totipotent state and eventually produce a complete individual. Abnormal reprograming is one of the main reasons for the low efficiency of SCNT (Loi *et al.* 2016); therefore, analysis of the key factors and regulatory mechanisms of abnormal development of SCNT embryos is an important way to improve the efficiency of SCNT. In the present study, we found that *lncRNA3720* was important for early embryonic development and SCNT reprograming, possibly by regulating key EGA genes and histone H2A variants.

EGA is an important process in early embryo development, and it was found to be deficient in SCNT embryos (Loi *et al.* 2016). Thus, we analyzed the key regulators in the EGA process to improve the efficiency of SCNT. Previous studies have shown that EGA occurs at the eight-cell stage in goats, which is characterized by significantly higher transcription levels than at other stages (Deng *et al.* 2019). We used RNA-seq to construct a transcript library of goat IVF-2c, IVF-8c and SCNT-8c embryos, and the sequencing data confirmed that the transcription level of IVF-8c embryos was higher than that of IVF-2c. The lncRNAs were highly expressed in early embryos, especially at the eight-cell stage, while the expression level in SCNT-8c embryos was lower than that in IVF-8c embryos. We screened 12 candidate lncRNAs during EGA in goat early embryos and determined the most highly expressed lncRNAs in IVF-8c embryos compared to IVF-2c and SCNT-8c. We identified one lncRNA (*lncRNA3720*) that may be a key promoter of embryonic development in goats. The role of histone variants during early development has been a major field in cell reprograming research, but further work is needed. The histone H2A family has more than 19 genes in humans and mice. Non-allelic variants include the species-conserved *H2A.X*, *macroH2A*, *H2A.Bbd* and *H2A.Z*. Several studies have reported that non-allelic histone *H2A* variants dramatically changed during genome remodeling in both IVF and SCNT embryos (Nashun *et al.* 2011, Wu *et al.* 2014, Duan *et al.* 2019); however, little is known about the canonical histone variants, *H2A1* and *H2A2*, as subfamily members in the early development of mammalian embryos. A previous study reported that histone H2A type 2-C was highly expressed in undifferentiated mammary epithelial cells and regulated oncogenic signaling (Monteiro *et al.* 2017). The pathways involved in the early embryonic development of the goat need to be further investigated.

With the development of high-throughput sequencing technology, the accumulated mammalian transcriptome data have been used to search for new functional genes. In the early development of mammals, more lncRNAs have been found by bioinformatics analysis to be involved in development and highly expressed at specific developmental stages (Li *et al.* 2016, Qiu *et al.* 2016, Arlic *et al.* 2017). However, due to the complexity of the regulatory mechanism of lncRNAs and the limitation of early embryo samples, the function of only a few lncRNAs have been characterized (Durruthy-Durruthy *et al.* 2016, Wang *et al.* 2016, Wang *et al.* 2018). Several studies have reported that lncRNAs can modulate gene expression during ZGA and was important for preimplantation development in mice (Arlic *et al.* 2017, Wu *et al.* 2018). Similarly, in goats, lncRNAs have been found to regulate stage-specific expression in early embryos and play vital roles during ZGA (Deng *et al.* 2018, Ling *et al.* 2019). At present, only one study reported the expression landscape of lncRNAs and that was in mouse SCNT embryos (Wu *et al.* 2018). The function and regulatory mechanism of lncRNAs in SCNT reprograming remain unclear. In this study, we found that lncRNA3720 was involved in early embryonic development and improved SCNT efficiency in goats, which emphasizes the importance of analyzing the function of lncRNAs for improving the reprograming of SCNT embryos.

In conclusion, we found that *lncRNA3720* was involved in early embryonic development in goats and affected the expression of the key EGA genes, *ZSCAN4* and *EIF1AX*, and the histone variant genes, histone *H2A* type 2-C and histone *H2B* type 1. Overexpression of *lncRNA3720* significantly improved the blastocyst rate and quality of SCNT embryos. Our findings highlight the critical roles of lncRNAs in promoting embryonic development and nuclear reprograming of SCNT and have great application value in the selection and breeding of superior goat breeds.

## Declaration of interest

The authors declare that there is no conflict of interest that could be perceived as prejudicing the impartiality of the research reported.

## Funding

This study was supported by National Natural Science Foundation of China (grant numbers 32072805) and Key Research and Development Projects of Shaanxi Province (grant numbers 2022NY-048).

## Author contribution statement

JMM analyzed the RNA-Seq data and performed experiments and wrote this paper. ZL designed the study and wrote the original draft. YHW performed WB and IF. ZJL performed microinjection. MHW created the graphs. CYZ collected sperm. ZJC provided resources. The other authors reviewed and approved the submit manuscript.
